# Prevalence of Overweight and Childhood Obesity on an Atlantic Island: A Cross-Sectional Study

**DOI:** 10.7759/cureus.103203

**Published:** 2026-02-08

**Authors:** Mariana Santos, Daniela A Valadão, Gonçalo Fonte, Inês Nunes, Isabel Silva, Beatriz Gonçalves, Rodrigo Baião, Irina Rodrigues, Beatriz Gomes, Tetiana Yahello, Oleksandr Yahello, Carlos Pestana

**Affiliations:** 1 Family Medicine, Centro De Saúde De Angra Do Heroísmo, Angra Do Heroísmo, PRT; 2 Family Medicine, Centro De Saúde da Praia da Vitória, Praia da Vitória, PRT

**Keywords:** azores, obesity, overweight, pediatric obesity, portugal

## Abstract

Background

Childhood overweight and obesity are major public health concerns, with limited recent epidemiological data available for the Azores region, particularly for Terceira Island. Accurate regional prevalence estimates are fundamental for the planning of effective prevention strategies. Therefore, this study aimed to determine the prevalence of overweight and obesity among children and adolescents on Terceira Island and to assess potential associations with age and sex.

Methods

An observational cross-sectional study was conducted between April and May 2025. The study population included individuals aged five to 18 years registered at Terceira Island’s primary health care facility. Participants were selected through simple random sampling, and data on age, sex, and body mass index (BMI) were extracted from clinical records. Corresponding BMI-for-age percentiles were calculated using standardized growth curves. Descriptive statistics and inferential analyses were performed to assess associations, including chi-square tests, Student’s t-tests, and logistic regression, with p-values <0.05 considered statistically significant.

Results

Of 680 individuals initially selected, 136 were excluded due to missing or outdated BMI records, resulting in a final sample of 544 participants (50.6% male). The prevalence of overweight was 41.2%, and obesity was 24.1%. Population-level prevalence estimates with 95% confidence intervals ranged from 37.2% to 45.2% for overweight and from 20.1% to 28.1% for obesity. No statistically significant associations were observed between these conditions and age or sex. These findings suggest a high prevalence of excess weight among children and adolescents on Terceira Island, regardless of age or sex.

Conclusion

Childhood overweight and obesity are highly prevalent on Terceira Island, and no differences were found by age or sex, highlighting the urgent need for early prevention and intervention strategies to address excess weight in this population.

## Introduction

The World Health Organization (WHO) defines overweight and childhood obesity as an excessive accumulation of body fat that poses a risk to health. Screening is performed using the WHO body mass index (BMI)-for-age growth reference curves, defining overweight as a BMI-for-age greater than one standard deviation above the WHO growth reference median and obesity as a BMI-for-age greater than two standard deviations above the WHO growth reference median, for children aged between five and 19 years [1].

Childhood obesity is associated with both immediate and long-term health consequences. Children with obesity are more likely to become adults with obesity and are at increased risk of developing noncommunicable diseases. In addition, obesity can lead to psychosocial problems, including low self-esteem, anxiety, depression, eating disorders, bullying, and a decline in school performance and quality of life [2-4].

According to the Childhood Obesity Surveillance Initiative (COSI) Portugal 2022, the prevalence of overweight among children aged six to eight years in Portugal was 31.9%, of whom 13.5% had obesity [5]. The Autonomous Region of the Azores reported the highest prevalence of overweight (43%) and obesity (22.8%), with a slightly higher prevalence in males (14%) compared to females [5]. A narrative review published in 2021 estimated that the prevalence of childhood obesity in Portugal ranges between 10% and 15%, and although some studies suggest a higher prevalence in females, most did not find statistically significant differences [6].

The implementation of strategies for early prevention, detection, and intervention is crucial to improve health outcomes, quality of life, and life expectancy in children and adults. To achieve this, healthcare professionals must understand the local epidemiology of the problem. Given the limited regional data, this study aimed to determine the prevalence of childhood overweight and obesity on Terceira Island, one of the nine islands of the Azores, and to assess potential associations with sex and age.

## Materials and methods

Study design

The study was designed as an observational cross-sectional study with the primary objective of estimating the prevalence of overweight and childhood obesity on Terceira Island and examining potential associations with age and sex. The data collection was conducted between April and May 2025.

Study setting

The study was conducted at the primary health care facility of Terceira Island - Unidade de Saúde da Ilha Terceira (USIT).

Study population and sampling

The study population comprised individuals aged five to 18 years registered at USIT at the time of data extraction (n=7095). Given the insular context and the organization of primary health care on Terceira Island, all resident children in this age group are registered at USIT, which provides universal primary care coverage for the island. ​​Participants were selected using simple random sampling, ensuring that each child had an equal chance of selection and thereby reducing selection bias and enhancing representativeness. Individuals without a recorded BMI within the past two years were excluded.

Sample

Sample size was calculated for a 95% confidence interval, assuming an estimated prevalence of 43% based on previous studies and a margin of error of 4%, selected as a balance between statistical precision and feasibility [5]. The following formula for estimating proportions in a finite population was used [7], where n is the required sample size, N is the population size (7095), p is the expected prevalence (0.43), d is the margin of error (0.04) and $Z_{1-\alpha/2}$ corresponds to the standard normal deviate for a 95% confidence level (1.96, α=0.05): $n=\frac{N p(1-p)}{\frac{d^2}{Z_{1-\alpha/2}^2}(N-1) + p(1-p)}$.

Sample size calculation was performed using OpenEpi (Version 3; an open-source epidemiologic calculator) (Atlanta, GA, USA) [8]. The calculated minimum sample size was 544. To enhance analytical robustness and account for an anticipated 20% exclusion rate, the target sample size was increased to 680 individuals. This approach ensured population-level prevalence estimates with acceptable precision.

Data collection

Data on age, sex, and BMI were extracted from clinical records in the MedicineOne® software (Life Sciences Computing S.A., Coimbra, Portugal) program by the investigators. BMI-for-age percentiles were determined using the WHO growth charts. All data were anonymized using alphanumeric codes to ensure participant confidentiality.

Variables

Overweight and obesity were defined according to the WHO BMI-for-age growth reference charts [1]. Overweight was defined as BMI-for-age greater than one standard deviation above the median, and obesity as BMI-for-age greater than two standard deviations above the median. Sex was categorized as female or male, and age was recorded in years.

Statistical analysis

Descriptive statistics were calculated for all variables. Associations between categorical variables were evaluated using the Chi-square test, and continuous variables were compared using Student's t-tests. Logistic regression analyses were also performed to explore potential associations between age and overweight or obesity as outcomes. A p-value <0.05 was considered statistically significant. Descriptive analyses were conducted using Microsoft Excel® (Microsoft Corp., Redmond, WA, USA), and inferential analyses were performed using IBM SPSS Statistics for Windows, version 30.0 (IBM Corp., Armonk, NY, USA).

Ethical considerations

The study protocol was reviewed and approved by the Ethics Committee of USIT (Approval number 03/2025, 24 April 2025). Data confidentiality was strictly maintained throughout the study, and no personally identifiable information was recorded. The authors declare no conflicts of interest.

## Results

A total of 7095 children aged five to 18 years were identified from USIT records. From this population, a simple random sample of 680 individuals was drawn, and after applying the exclusion criteria, the final sample comprised 544 participants, including 275 males (50.6%) and 269 females (49.4%). The mean age was 12.1±4.0 years (range 5-18 years). The observed prevalence of overweight and obesity in the sample, as well as the estimated prevalence for the population, are presented in Table 1. Population prevalence was estimated to provide a more accurate and generalizable measure and ensure the public health relevance of the findings.

**Table 1:** Prevalence of overweight and obesity Overweight and obesity were classified according to the WHO BMI-for-age growth reference standards [1]. CI: confidence interval; BMI: body mass index; WHO: World Health Organization

Variable	Frequency (n)	Sample prevalence (%)	Population prevalence (95% CI)
Overweight	224	41.2	37.2%-45.2%
Obesity	131	24.1	20.1%-28.1%

Associations between sex and childhood overweight and obesity were evaluated using the Chi-square test. No statistically significant associations were observed for either condition (p>0.05), indicating that prevalence did not differ between males and females in this sample. Also, associations between age and overweight and obesity were assessed using Student’s t-test and logistic regression. No statistically significant associations were found for either condition (p>0.05), indicating that the prevalence of these conditions did not vary meaningfully with age in this sample.

## Discussion

This study is the first to investigate childhood overweight and obesity on Terceira Island, providing locally relevant data for a region previously reported as having the highest prevalence of the conditions in Portugal [5]. The observed prevalence of overweight (41.2%) and obesity (24.1%) aligns with COSI study 2022 data for the Azores (43% for overweight and 22.8% for obesity) and is slightly higher than national estimates from a 2021 narrative review, which reported ranges of 20% to 40% for overweight and 10% to 15% for obesity [6].

Compared with other European countries, the prevalence identified in this study exceeds typical childhood overweight and obesity rates, which generally range between 20% and 35% [9-12]. Evidence from Spain and Norway suggests that childhood overweight often persists into adolescence, with more than half of affected children maintaining excess weight over time [10,11].

No statistically significant associations were observed between sex and overweight or obesity, consistent with previous findings [5,6] reporting either inconsistent or non-significant sex differences, although national surveillance data indicate a slightly higher prevalence among males [5]. This lack of association in the present study may reflect relatively homogeneous exposure to obesogenic environments across sexes in this regional context. Similarly, no significant differences were found in mean age between children with and without overweight or obesity, and logistic regression did not indicate an association with age. This may be explained by the cross-sectional design, which limits the ability to detect temporal trends, or by the early onset and persistence of excess weight throughout childhood and adolescence. Nonetheless, potential associations at the population level cannot be excluded.

Several limitations of this study should be acknowledged. The cross-sectional design precludes causal inference and limits the assessment of temporal trends. Additionally, reliance on clinical records may introduce measurement bias, as height and weight data could have been inaccurately recorded or outdated. Despite these limitations, the study benefits from several strengths, including simple random sampling to minimize selection bias, a large and representative sample, and the use of standardized WHO growth references, enhancing the reliability and comparability of the findings.

Further research is needed to explore the determinants of childhood overweight and obesity in this population, considering the Azores' unique geographic, socioeconomic, and cultural context. Family-related factors, such as parental nutritional status, genetic predisposition, and education, are important contributors [13,14]. Breastfeeding, which has a protective effect against obesity, is less prevalent in the Azores, where exclusive breastfeeding rates are among the lowest in Portugal [15,16]. Childhood dietary habits, physical activity, sleep patterns, and the school environment also play critical roles in shaping long-term body weight trajectories [17,18].

The high prevalence observed underscores that childhood overweight and obesity remain a significant public health challenge in the Azores, requiring targeted and context-specific interventions. Family physicians play a key role in addressing these conditions through health promotion, preventive care, early detection, and longitudinal follow-up. Their long-term relationships with families and communities enable the identification of children at risk, the promotion of healthy lifestyles, parental education, and collaboration with schools and local stakeholders. Interventions such as nutrition and cooking programs, organized sports, and school-based initiatives may be effective in fostering healthy behaviors. Early identification and prevention are particularly relevant in regions such as the Azores, where the burden of obesity-related morbidity may have substantial implications for population health.

## Conclusions

The estimated prevalence of overweight among children and adolescents on Terceira Island ranged from 37.2% to 45.2% (95% CI), while obesity prevalence ranged from 20.1% to 28.1% (95% CI). These high rates highlight a significant public health concern and underscore the urgent need for effective, locally adapted strategies for prevention, early detection, and intervention. Family physicians are well-positioned to lead these efforts by promoting healthy lifestyles and supporting early and sustainable interventions.
